# Bioprocess Engineering for Enhanced Zinc Solubilization Through Response Surface Methodology (RSM) by Newly Isolated Rhizobacteria

**DOI:** 10.3390/microorganisms14102286

**Published:** 2026-10-08

**Authors:** Mohd Ashraf Dar, Ebru Koca Akkaya, Ommer Bhat, Mahdi Hassan, Garima Kaushik

**Affiliations:** 1 Department of Environmental Engineering, Yildiz Technical University, Davutpasa, Esenler, Istanbul 34220, Türkiye; 2 Department of Environmental Science, School of Earth Sciences, Central University of Rajasthan, BandarSindri, Ajmer 305817, Rajasthan, India

**Keywords:** Zn-solubilizing bacteria, *Serratia marcescens*, Zn-solubilization, optimization, Box–Behnken design

## Abstract

Zinc (Zn) is an essential micronutrient for optimum plant growth; however, Zn deficiency is widespread in agricultural soils due to the low availability of plant-accessible or soluble forms of Zn. Therefore, harnessing microorganisms’ ability to solubilize Zn can be a significant, environmentally beneficial contribution to sustainable agriculture. This study isolated Zn-solubilizing bacteria (ZnSB) from rhizosphere soil and assessed their capacity to solubilize Zn minerals (ZnO, ZnCO_3_). Based on qualitative and quantitative evaluation, the highest solubilization efficiency (345%) and 954 ± 46 mg Zn L^−1^, respectively, were observed in the AEYTU11 strain and were associated with a strong pH decrease in the culture medium. Based on phenotypic characterization and 16S rRNA sequence analysis, strain AEYTU11 was identified as *Serratia marcescens*. The in vitro solubilization potential of microbes is significantly influenced by process parameters, emphasizing the importance of optimizing the culture conditions to maximize microbial solubilization efficiency. A Box–Behnken design (BBD) experiment with 17 combinations of varying levels of insoluble Zn compound (0.5–1.5 g/L), pH (5–9), and incubation time (3–5 days) was used to model their effects on *Serratia marcescens* AEYTU11 strain-mediated Zn release from ZnCO_3_. Following process optimization, the solubilization efficiency of the AEYTU11 strain increased, achieving a maximum Zn solubilization capacity of 1608 ± 51 mg/L. The resulting model had high predictive ability (R^2^ = 0.9099) and showed that ZnCO_3_ concentrations were the most significant factor for the amounts of Zn released. In addition to Zn solubilization, AEYTU11 possess potassium (K) solubilization and extracellular enzyme production for protease and cellulose activity. The optimization findings highlight the value of statistical optimization tools for maximizing microbial Zn-solubilizing activity and improving biofertilizer development.

## 1. Introduction

Micronutrient (MN) availability significantly affects plant growth and development (PGD), maturity, and yield. Zn is one of the key MN that is vital for cell metabolism throughout PGD [1]. For optimal plant growth, Zn is needed in tissues at relatively low concentrations (5–100 mg kg^−1^) [2], yet in many regions of the world, a Zn deficit has become a limiting factor for agricultural yield [3]. One of the main causes of human malnutrition due to Zn deficiency is the wide variety of agronomic soils used for grain cultivation worldwide that are deficient in Zn [4]. Zn deficiency is thought to be the most common abiotic stress among MN deficiencies in several nations and regions, including Asia, Australia, Türkiye, Europe, the United States, and Africa [3,5]. This is because over 2800 proteins require Zn for both structural integrity and function [6], and Zn is the only MN that is pertinent to all kinds of enzymes found in biological systems [7]. Reduced membrane integrity and decreased synthesis of carbohydrates, auxins, nucleotides, cytochromes, and chlorophyll result from Zn deficiency in plants, which also makes them more vulnerable to heat stress [8]. In addition to lowering crop yields, a Zn deficit in millions of hectares of agricultural soil has seriously compromised the nutritional value of the food products, leading to serious health and nutritional issues for one-third of the world’s population [4,9]. Overuse of Zn fertilizers can also potentially harm people by impairing their ability to absorb iron and copper and suppressing male sexuality as well [2]. Seemingly, increasing the pool of Zn in the soil by applying organic manures or synthetic fertilizers containing Zn becomes essential. Zn EDTA or Zn sulfate, two synthetic fertilizers, appear to be a quick and simple means to mend Zn deficiencies, but are a non-sustainable and uneconomical approach [10]. Crops need roughly 0.05 kg ha^−1^ Zn per ton of grain output, but Zn is not readily available to the plant; so larger doses of fertilizer are needed [11]. Unfortunately, 96–99% of applied Zn is again converted into inaccessible Zn pools by precipitation to carbonates, oxides, phosphates, etc.; therefore, exogenous use of chemical fertilizers alone cannot effectively battle soil Zn deficiency over the long run [3,12]. Farmers typically use a lot of chemical fertilizers, which can have a negative impact on the environment and human health as well [11,13]. Furthermore, the majority of Zn fertilizers dissolve in soil somewhat slowly, often at a rate that is insufficient to provide essential levels of Zn for PGD [14]. Therefore, reduced chemical Zn fertilizer usage efficiency is a problem, particularly over the long run.

Exploiting the natural ability of some soil microorganisms, particularly bacteria, to solubilize the fixed forms of Zn into labile forms for increased accessibility and subsequent uptake by plants would be a viable and sustainable alternative to reduce Zn fertilizers inputs into the environment and make effective use of the natural soil Zn reservoirs. A significant amount of Zn is present in soils in fixed forms such as franklinite, zincite, sphalerite, smithsonite, wellemite, and hopeite, which are sparingly soluble [3]. The accessible soil Zn level is quite low (4–270 μgL^−1^) compared to the 64 mg kg^−1^ mean total Zn level, according to values reported in the literature [5]. Therefore, using bacteria that can convert the fixed forms of Zn found in soil into accessible forms to meet plant Zn needs would be an alluring alternative. Although several ZnSB have been isolated from the soils of diverse zones, such as *Pseudomonas protegens* [10], *Agrobacterium tumefaciens* and *Rhizobium* sp. [11], *Pantoea eucrina* [15], *Bacillus megaterium* [3], *Serratia nematodiphilia* and *Acinetobacter pitti* [16], etc., capable of solubilizing Zn. However, the chemical and biological intricacy of the process limits our understanding of how microorganisms convert insoluble Zn to a soluble form. However, several researchers have proposed that sugar oxidation into organic acids is the first step in the bio-conversion of chemical compounds. This lowers the medium’s pH and improves the solubilization of insoluble Zn [17]. Additionally, *Bacillus megaterium*’s capability to solubilize ZnO, ZnCO_3_, and Zn_3_(PO_4_)_2_ was linked by Dinesh et al. [3] to a significant drop in pH because of increased gluconic acid generation. The results of Khande et al. [18] also support the idea that gluconic acid generation causes increased Zn solubilization at lower pH.

Numerous researchers have also investigated the various elements, including growth and process conditions, that can affect conversion efficiency [19]. Depending on the microbial species, different nutrient media have different compositions. Consequently, one of the most important aspects of employing microbial species to solubilize insoluble Zn compounds is optimizing the growth conditions [20]. Traditional approaches of examining how many parameters affect growth conditions and the solubility of insoluble chemicals by microbes, with only one variable held constant and other aspects remaining constant, are costly and time-consuming [21]. Furthermore, there is no assurance that the employed approach will be able to identify the ideal circumstances for the targeted microorganism’s metabolic activity [22]. To determine the ideal growth conditions for microorganisms to achieve optimum competence in bioactivities, including insoluble Zn compound solubilization, the response surface method (RSM) can be a useful substitute in this regard [21]. To develop a predictive mathematical model that might assess the influence of multiple factors acting as independent variables on an ideal response acting as a dependent variable, these techniques integrate statistical and mathematical approaches for experiment design [23]. These techniques predict or optimize the response variable by combining quantitative data from a suitable number of tests and integrating them through the analysis of multi-parameter equations.

Given the facts, to the best of our knowledge, studies specifically addressing the isolation, molecular identification, and Zn solubilization capacity of indigenous bacterial isolates from Turkish soils are scarce or absent in the peer-reviewed literature. To fill this gap, the current study aimed to isolate and identify a novel ZnSB from Türkiye’s soils for the first time. Along with their ability to solubilize Zn, the shortlisted ZnSB strains were also evaluated for their multitasking capabilities, including P and K solubilization, production of IAA, ammonia (NH_3_), hydrogen cyanide (HCN), siderophores, and cell wall-degrading enzymes. Furthermore, this study sought to optimize the Zn solubilization process of the most potent strain using an RSM model to elucidate its effect on Zn solubilization.

## 2. Materials and Methods

### 2.1. Isolation of ZnSB

Soil samples from the rhizosphere of *Hydrangea macrophylla* at Yildiz Technical University, Istanbul province (Turkiye), were collected for the isolation of ZnSB. Soil that is within limits of area inhabited by plant roots and that clings firmly to roots was considered to be rhizospheric soil [24]. A total of five individual rhizosphere soil samples were collected using a random sampling strategy during October 2025 by gently exposing the fine active root zone with a sterile trowel. These samples were thoroughly combined to prepare a single homogeneous composite rhizospheric soil sample, which was immediately processed under aseptic conditions for the isolation of ZnSB. Briefly, 1 g of soil was added to 9 mL of sterile distilled water. The suspension was then serially diluted up to 10^−7^ after being agitated and left undisturbed for five minutes. Aliquots of 100 μL from dilutions 10^−3^ to 10^−7^ were inoculated onto basal Zn-solubilizing medium (ZnSM) (gL^−1^ glucose-10; (NH_4_)2SO_4_-1; KCl-0.2; K_2_HPO_4_-0.1; MgSO_4_-2; and agar-15), supplemented with 1 gL^−1^ of insoluble Zn compound (ZnO), incubated for 3 days at 32 ± 2 °C to evaluate their Zn solubilization capability [25]. Bacterial colonies that showed clear zones surrounding their colonies on ZnSM agar Petri plates were identified as ZnSB and were subjected to additional purification on nutrient agar plates. Following purification, each strain was kept for further experiments in a 20% glycerol stock at −20 °C.

### 2.2. Qualitative Zn-Solubilizing Assay of Potential Strains

To evaluate the isolated strains’ capacity to solubilize insoluble Zn compounds qualitatively. Separately, 0.1% insoluble Zn from two sources: ZnO and ZnCO_3_, was added to the basal medium (ZnSM). The Petri plates were point-inoculated with one loopful of an overnight culture of bacterial isolates, and they were then incubated for 10 days at 32 ± 2 °C. After 10 days, the zone of solubilization/clearance was measured and reported in mm. Using the following formulas, the halo zone was measured to calculate the strains’ Zn solubility index (*ZnSI*) and solubilization efficiency (*ZnSE*) [26].

(1) $$ZnSI=\frac{HzD+CD}{CD}$$

(2) $$ZnSE=\frac{HzD+CD}{CD}\times 100$$

where *HzD* denotes solubilization halo zone diameter, and *CD* is colony diameter.

### 2.3. Identification of Potent ZnSB

The most promizing shortlisted AEYTU11 strain was identified via 16S rRNA gene sequencing. AEYTU11 was cultured overnight, and bacterial genomic DNA was extracted [27,28]. Its quality was evaluated on a 1.0% agarose gel, and a single band of high-molecular-weight DNA was observed. A fragment of the 16S rRNA gene was amplified by 16SrRNA-F and 16SrRNA-R primers. A single discrete PCR amplicon band of 1500 bp was observed when resolved on an agarose gel. The PCR amplicon was purified to remove contaminants. Forward and reverse DNA sequencing reactions of the PCR amplicon were carried out with 16SrRNA-F and 16SrRNA-R primers using the BDT v3.1 cycle sequencing kit on an ABI 3730xl genetic analyzer by Sree Biologix, Hyderabad, India. The consensus sequence of the 16S rRNA gene was generated from forward and reverse sequence data using aligner software (BioEdit Version 7.2.5).

The BLAST program was used to perform sequence analysis, which was then compared to registered sequences in the GenBank database (http://www.ncbi.nlm.nih.gov/BLAST), and the highest sequence homology score was used to identify the species. Thirteen 16S rRNA gene sequences that closely matched the strain were obtained from GenBank, and the CLUSTALW program (version 1.6) was used to align the sequences. Using 1000 repeats and the neighbour-joining approach with MEGA-12, assisted by bootstrapping, a phylogenetic tree of 16S rRNA sequences was produced [29]. In summary, CLUSTALW integrated in MEGA-12 was used to align the sequences of various bacteria described in the literature with the sequence of the best Zn-solubilizing strain, AEYTU11.

### 2.4. Quantitative Zn-Solubilizing Assay

The ZnSB that displayed the highest *ZnSI* underwent additional testing to determine whether it could release inorganic Zn in a liquid medium. The AEYTU11 was first pre-cultivated in LB medium overnight before being pelleted by centrifugation (6000 rpm, 15 min). The cells were rinsed twice with sterile water, and 1 mL of cell suspension was used as the inoculant for the quantitative test. A 250 mL flask was filled with 100 mL ZnSM without agar that had been supplemented with 0.1% insoluble Zn, individually from two sources: ZnO and ZnCO_3_. 1 mL suspension of a single bacterial culture was then added to the flasks and maintained for 5 days at 120 rpm (32 ± 2 °C) in an incubator-shaker. Each experiment contained three replicates to reduce error, and the controls were uninoculated flasks with comparable concentrations of the corresponding Zn sources. Representative samples (10 mL) were aseptically taken on the 5th day, and the bacterial strain’s viability was verified by streaking on agar plates. The change in culture pH and uninoculated medium after 5 days was also determined in parallel via a WTW pH 340i meter to confirm the release of organic acids. The mixture was aseptically shifted to tubes and spun at 8000 rpm (15 min), and the collected supernatant was subsequently filtered via a 0.22 μm syringe filter to acquire a culture filtrate with soluble forms of Zn only. Following filtration, samples were analyzed for soluble Zn concentrations via a PerkinElmer Optima 2100 DV inductively coupled plasma–optical emission spectrometer (ICP–OES). The quantity of solubilized Zn was determined by subtracting the soluble Zn concentration in the uninoculated control from that in the inoculated sample, with the result expressed as mg Zn L^−1^.

### 2.5. Phenotypic and Biochemical Characterization

Phenotypic and biochemical traits of the most promising ZnSB, including Gram reaction, KOH Stirring, motility, spore formation, H2S production, indole, urease, methyl red, hemolysis, lactose, citrate, and catalase activity, were evaluated with the methodologies described by Tindal et al. [30].

#### 2.5.1. Indole-3-Acetic Acid (IAA) Production

Quantification of IAA production by the AEYTU-11 was performed following the protocol outlined by Dinesh et al. [3]. Briefly, a 50.0 μL aliquot of the bacterial cell suspension was inoculated into 15 mL culture tubes containing 5 mL of sterile tryptone yeast extract broth (comprising g L^−1^; tryptone-10, beef extract-3, NaCl-5, L-tryptophan-0.204). The cultures were incubated in the dark at 30 °C for 3 days. Subsequently, a 2 mL aliquot of culture broth was centrifuged at 8000 rpm for 7 min. 1 mL resulting supernatant was transferred to a new 2 mL microcentrifuge tube and reacted with 1 mL of freshly prepared Salkowski reagent (50 mL HClO_4_ (35%), 1mL 0.5 M FeCl_3_ solution). The mixtures were then incubated in the dark at 30 °C for 1 h. The development of a distinct red coloration within the medium served as a positive indicator of IAA biosynthesis.

#### 2.5.2. Hydrogen Cyanide (HCN) Production

Qualitative assessment of HCN production was conducted using the modified method of Kloepper et al. [31]. AEYTU-11 strain in its logarithmic growth phase (25 μL) was inoculated into 30 mL sterile glass vials containing 5 mL of King’s B broth supplemented with glycine (4.4 gL^−1^). Filter paper strips pre-soaked in an alkaline picric acid solution (gL^−1^ picric acid-2.5 + Na_2_CO_3_-12.5) were suspended in the headspace of the vials, ensuring no direct contact with the liquid medium. The vials were hermetically sealed with screw caps and parafilm and subsequently incubated under continuous agitation in a mechanical shaker for 3 days. A colorimetric transition of the filter paper strips from yellow to brown or red indicated the volatile production of HCN.

#### 2.5.3. Phosphate and Potassium Solubilization

Phosphate (P) and Potassium (K) solubilization efficiencies were evaluated by spot inoculating a loopful of a 24 h broth culture onto Pikovskaya’s agar medium [32] and Aleksandrov agar medium [33]. The plates were examined visually after three days of incubation at 30 °C, and the clear zone of solubilization indicated the strain’s P and K solubilization activity.

#### 2.5.4. Production of Hydrolytic Enzymes

The secretion of extracellular cell-wall-degrading enzymes (α-amylase, cellulase, and protease) was assayed using the methodologies described by Cappuccino and Sherman [34]. A loopful of bacterial suspension was line-streaked onto starch agar plates (gL^−1^; peptone-5, beef extract-3, soluble starch-10, agar-15) and skim milk agar plates to measure α-amylase and protease activity. Plates were assessed for the presence of a transparent zone of hydrolysis surrounding the bacterial growth line following a 48 h incubation period at 30 °C.

For cellulolytic activity, bacteria were streaked over a basal medium supplemented with 1% (*w*/*v*) cellulose. Following three days of incubation at 30 °C, the agar surface was flooded with 0.01% (*w*/*v*) Congo red for fifteen minutes, and then it was destained for five minutes using a 1% (*w*/*v*) NaCl solution. Cellulase enzymatic activity was verified by a clear zone of clearing against an opaque red background [3].

### 2.6. Experimental Design and Optimization via RSM

The impact of important process factors, such as pH, incubation duration, and the starting insoluble Zn content, on the efficiency of bacterial Zn solubilization was optimized and assessed using RSM in conjunction with a Box–Behnken Design (BBD). As shown in Table 1, the experimental design included three independent factors at three levels (−1, 0, and +1), with an initial broth pH of 5.0 selected as the lower boundary in the BBD matrix to evaluate bacterial adaptation and growth starting in slightly acidic conditions. The Zn solubilization efficiency of the possible bacterial strain (AEYTU-11) was characterized using a complete quadratic model with 17 experimental runs derived from 12 factorial points and 5 center points (used for determining experimental error). The total number of experimental runs was determined using Equation (3), as reported by previous studies [35,36].

(3) $$N\;=\;2K\;(K\;-\;1)+\;C_{0}$$

where *K* and $C_{0}$ represents the number of factors and center points, respectively. The experimental results were subsequently fitted to a second-order model to elucidate the relationship between the process variables and the Zn solubilization efficiencies by the bacteria. This model accounts for the linear, quadratic, and interaction effects of the independent variables. The general mathematical expression of the model is presented in the following Equation (4).

(4) $$Y=\beta_{0}\;+\sum_{j=1}^{K}\beta_{j}X_{j}+\sum_{j=1}^{K}\beta_{jj}X_{j}^{2}\;+\sum_{i}\;\sum_{<j=2}^{K}\beta_{ij}X_{i}X_{j}\;+\;e_{i}$$

In the model, *Y* correspond to the response, while *Xi* and *Xj* denote independent variables ranging from 1 to *k*. β_0_ corresponds to the model intercept coefficient, whereas *β_j_, β_jj_*, and *β_ij_* represent the coefficients of the linear, quadratic, and interaction terms, respectively. Here, *k* denotes the number of independent variables (*k* = 3), and *e_i_* represents the experimental error.

The developed models were statistically validated using analysis of variance (ANOVA), based on the F-test and corresponding *p*-values for the response. Adequacy and goodness-of-fit of the models were further assessed using the coefficient of determination (R^2^), adjusted (R^2^), and adequate precision values. Additionally, two-dimensional contour plots (2D) and three-dimensional (3D) response surface plots were generated to identify the optimal interaction effects and operating regions of the process variables. All statistical analyses were conducted using the trial version of the Design-Expert software package developed by Stat-Ease 360 Inc., Minneapolis, MN, USA.

## 3. Results and Discussions

### 3.1. Isolation and Qualitative Zn Solubilization

Samples of rhizospheric soil were serially diluted and applied to a base medium that contained 0.1% insoluble ZnO. From this enrichment culture, three morphologically different isolates exhibiting Zn-solubilization traits were recovered. We used ZnSM agar plates containing two insoluble Zn sources (ZnO, ZnCO_3_) to qualitatively assess these strains’ ability to solubilize Zn. The bacterial strains showed variable degrees of solubilization across two Zn compounds, as indicated by variations in halo zone formation (Table 2). Dissolution zones were markedly larger with ZnO, followed by ZnCO_3_, for AEYTU-13 and AEYTU-15, while for AEYTU-11, a larger zone of solubilization was observed for ZnCO_3_ (Figure S1). After 10 days of incubation, AEYTU-11 showed the highest solubilization index of 2.71 and 3.45 with ZnO and ZnCO_3_, and efficiency of 271% and 345%, followed by ZnSB2 (260 and 233% with ZnO and ZnCO_3_, respectively). The results indicate that the efficiency varied both between bacterial strains and the Zn source applied. However, the literature reports show contradictory results, with greater solubilization zones observed with different compounds depending on carbon source (CS) and experimental conditions [3,10,11]. The halo formation signified solubilization zones attributed to pH lowering and consequent dissolution of insoluble Zn compounds near the bacteria. The size of the dissolution zone, however, has been reported to vary depending on the CS (sucrose/glucose) used in the assay [37]. A larger solubilization zone has been noted using ZnO [3], Zn_3_(PO_4_)_2_ [38], or ZnCO_3_ [39]. Different *ZnSI* and *ZnSE* were found in isolates in the current investigation, with ranges of 2.25 to 3.45 and 225 to 345%, respectively. These findings are corroborated by the study of Mumtaz et al. [40] and Ramesh et al. [41], which reported *ZnSE* up to 296% and 175% by *Bacillus aryabhattai*, respectively. However, in our situation, the maximum solubilizing efficiency was observed for AEYTU-11 when ZnCO_3_ was used as an insoluble Zn source. However, Gandhi and Muralidharan [42] observed that *Acinetobacter* sp. (AGM3) exhibited the maximum Zn solubilization in ZnO-enhanced medium, reaching 36.54 mg mL^−1^. Because of its greater solubilization capability, strain AEYTU-11 was chosen for further in-depth investigations of Zn solubilization in this study.

### 3.2. Identification of AEYTU-11 Strain

A phylogenetic tree (Figure S2) illustrating the AEYTU-11 strain’s position within the *Serratia* genus was generated using the MEGA-X program (Version-10.1.2). These findings led to the designation of the AEYTU-11 strain as *Serratia marcescens*, as a BLAST search of the partial 16S rRNA gene sequence against the NCBI GenBank database demonstrated high sequence identity (>99%) and 100% query coverage with reference strains belonging to the *Serratia marcescens* group (Table S1). The NCBI GenBank database now contains the nucleotide sequence with accession number PZ502334. *Serratia* sp. is a versatile bacterium with the ability to solubilize different insoluble nutrients and has been reported to solubilize P [43,44], K [45], iron, and Zn [46]. *Serratia* sp. has also been reported to degrade a range of pollutants, including the phenolic pollutant p-cresol [47], diazinon [48] and dyes [49]. While members of the genus *Serratia* are widely recognized in the literature as effective nutrient-solubilizing agents as well as bioremediation candidates, their biosafety considerations must be carefully addressed due to opportunistic clinical associations. In this study, the isolated *Serratia* strain underwent preliminary biosafety screening via blood agar assays, exhibiting gamma (γ) hemolysis (non-hemolytic phenotype), indicating the absence of red blood cell lysis capacity. However, we explicitly acknowledge that available biosafety data are preliminary and insufficient to establish strain safety. Substantial additional genomic and in vivo toxicological assessments are imperative before evaluating the strain for agricultural deployment. To the best of our knowledge, no previous study has reported the identification of an indigenous ZnSB from Türkiye. Furthermore, although Zn solubilization has been documented for several bacterial genera worldwide, studies specifically investigating Zn solubilization by *Serratia marcescens* remain limited. Therefore, the present study represents the first report of Zn-solubilizing S. marcescens isolated from Türkiye and contributes information to the understanding of bacterial Zn solubilization.

### 3.3. Morphological, Biochemical, Enzymatic, and Nutrient-Solubilization Traits of AEYTU-11 Strain

AEYTU-11 strain was observed as a red, Gram-negative, non-spore-forming, motile, rod-shaped bacterium with convex elevation and smooth margin, generating circular colonies on NA plates (Figure S3). Among the biochemical characteristics evaluated, AEYTU-11 exhibited negative reactions for H_2_S production, indole, urease, and methyl red, while positive activities for lactose, citrate, and catalase reactions (Table S2). *Serratia marcescens* is widely documented in the literature as a potential auxin source [50,51]; however, strain AEYTU-11 demonstrated a negative response for IAA production under the tested conditions. This variation underscores the strain-specific nature of secondary metabolic pathways in *S. marcescens*, which can be heavily influenced by precursor availability or potential phenotypic variation during in vitro cultivation. Assessment of hydrolytic enzyme activities revealed that the AEYTU-11 strain was positive for protease production and cellulose activity, while negative for α-Amylase activity (Table S2). Nutrient solubilization traits revealed that P solubilization ability was absent in AEYTU-11, while it demonstrated positive K-solubilizing ability. Regarding biocontrol-associated traits, hydrogen cyanide (HCN) production was also detected in AEYTU-11.

### 3.4. Quantitative Assay for Zn Solubilization

The plate assay does not accurately reflect the bacterial strains’ ability to solubilize or mineralize insoluble substances [52]. The *Serratia marcescens* AEYTU-11 strain was further tested in a liquid medium (broth assay) separately containing two different Zn sources, including ZnO and ZnCO_3_. Regarding ZnO, the highest amount of accessible Zn recorded by AEYTU-11 was 352 ± 17 mg L^−1^ and 966 ± 46 mg L^−1^ for ZnCO_3_ on the 5th day. However, there were meagre variations in the amount of Zn dissolved in the control treatments (29.8 ± 1.4 mg L^−1^ and 12.2 ± 0.6 mg L^−1^ for ZnO and ZnCO_3_, respectively). Therefore, the net Zn solubilized (difference between the available Zn content in AEYTU-11 broth and the available Zn content in the control) was 322 ± 17 and 954 ± 46 mg L^−1^ when ZnO and ZnCO_3_ were used as insoluble Zn sources, respectively. The AEYTU-11 strain’s capacity to solubilize Zn was validated by a qualitative analysis, which showed that ZnCO_3_ had the highest Zn solubilization, followed by ZnO, which was consistent with the quantitative results. These findings concur with those of Yasmin et al. [10] and Dinesh et al. [3]. *Bacillus* sp. and *Pseudomonas* sp. were evaluated by Saravanan et al. [25] for their capacity to solubilize Zn in both plate and broth experiments. They discovered that the bacterial isolates’ solubilization potential differed. On the 15th day, their findings showed increased Zn solubilization, with 13.6 mg kg^−1^ of Zn in ZnS, 16.4 mg kg^−1^ in ZnO, and 13.4 mg kg^−1^ in ZnCO_3_, respectively. The highest Zn solubilization by *Acinetobacter* sp. was observed in both ZnO and ZnCO_3_ growth media, with values of 36.5 and 35.4 µg Zn mL^−1^, and 33.14 and 32.69 µg Zn mL^−1^, respectively, by Gandhi and Muralidharan [42]. In contrast to Ramesh et al. [41], who showed that when *B. aryabhattai* strains were inoculated, the solubilization of Zn was higher in a medium enriched with Zn_3_(PO_4_)_2_ than in ZnO or ZnCO_3_. The medium’s pH significantly decreased along with the increased labile Zn content, indicating that AEYTU-11 generated organic acids that lowered the pH and aided in the dissolution of the more insoluble Zn compounds. Acidification is one of the ways that Zn-soluble bacteria solubilize the metals. These microorganisms generate organic acids that lower pH by sequestering Zn cations [15,53]. In our study, a sharp decrease in culture broth pH was consistently observed alongside peak Zn release, confirming that organic acid production and proton-mediated dissolution may serve as the primary mechanisms responsible for solubilizing Zn complexes by the *Serratia marcescens* AEYTU-11 strain. The results of Dinesh et al. [3] and Khande et al. [18] support the idea that gluconic acid generation causes increased Zn solubilization at lower pH. The pH of the AEYTU-11-treated assay decreased noticeably in comparison to the control, from 7.2 (day 0) to 5.64 and 2.92 on the 5th day for ZnO and ZnCO_3_, respectively. In actuality, AEYTU-11 recorded the maximum amount of soluble Zn (954 ± 46 mg L^−1^) on the 5th day when the pH was 2.92. As the *Serratia marcescens* AEYTU-11 strain exhibited the highest Zn solubilization efficiency in the presence of ZnCO_3_ compared with ZnO, ZnCO_3_ was selected as the Zn source for subsequent optimization experiments.

### 3.5. Experimental Design and Optimization via RSM

**Figure 1:**
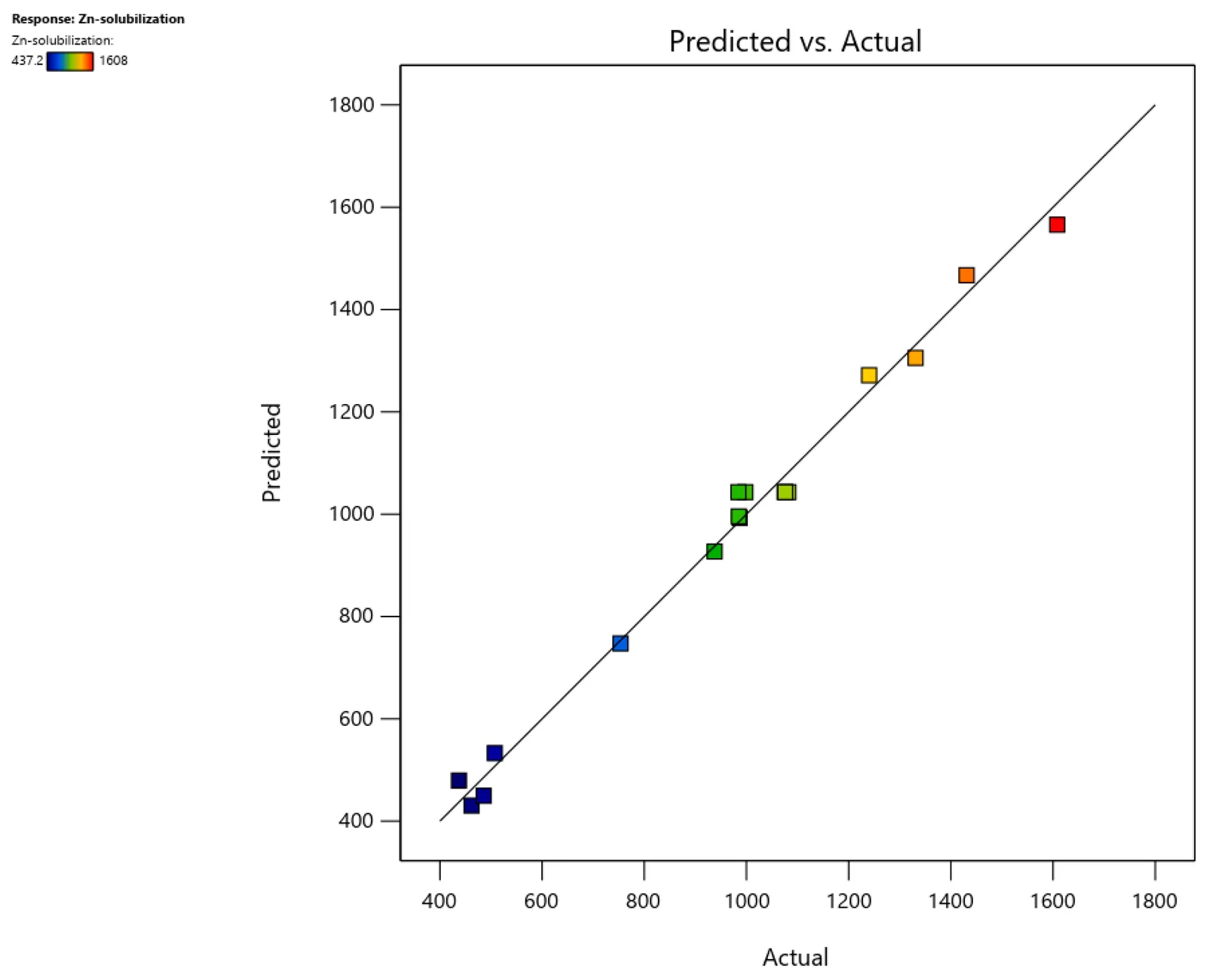
Relationship between predicted and actual values for Zn-solubilization by AEYTU-11.

RSM is a process development, improvement, and optimization method that combines statistical and mathematical techniques. Even when complex interactions are present, it is utilized to assess the relative importance of many influencing elements. Typically, RSM consists of three steps: (1) design and experimentation; (2) regression-based response surface modelling; (3) optimization [54]. To design the tests and assess the impact of independent factors on Zn solubilization, BBD, a well-liked second-order experimental design, was employed. When used for sequential experimentation, BBD is an efficient design that reduces the total cost of the experiment by requiring a comparatively small number of design points and offering a sufficient amount of information for verifying the goodness of fit [55]. RSM based on a BBD was employed to evaluate the effects of three independent variables on Zn solubilization. The experimental design incorporated five center points, resulting in a total of 17 experimental runs. The BBD matrix, presented in Table 3, includes both the actual and coded levels of the independent variables, together with the corresponding experimental and model-predicted responses. The effects of the individual variables as well as their interaction terms on the response were systematically investigated. As shown in Table 3, the predicted values from the developed models closely agreed with the experimental results for Zn solubilization. This relationship is further supported by the diagnostic plot (Figure 1), where the data points are distributed near the diagonal, indicating a strong correlation between the observed and predicted responses and demonstrating the adequacy of the developed model [35,36].

The second-order response variable, Zn solubilization by *Serratia marcescens*, was modelled using multiple regression analysis. Statistical significance was evaluated using a threshold *p*-value of <0.05 to identify the factors affecting Zn solubilization. The quadratic Equation (5) was developed to determine the interactive correlation among response and process factors. The resulting final model in terms of coded factors is given below.

Zn-solubilization (Y) = 1043 + 464.71A − 44.11B − 78.48C − 53.67AB − 51.70AC − 45.65BC − 41.56A^2^

−96.76B^2^ − 30.54 C^2^ (5)

The direction and severity of the independent variables’ effects on Zn solubilization (Y) are shown by the sign and magnitude of the coefficients in Equation (5). ZnCO_3_ concentration (A, +464.71) showed the strongest positive influence among the relevant parameters, suggesting that increases in the A parameter improved Zn solubilization by *Serratia marcescens*. On the other hand, the pH quadratic term (B^2^ − 96.76) had a substantial negative impact, indicating the existence of diminishing returns at higher levels.

ANOVA was employed to evaluate the BBD experimental data utilizing F and *p*-values, as presented in Table 4. The high F-values and low *p*-values (*p* < 0.0001) obtained for the developed mathematical model indicate that the model is obviously significant and adequately describes the experimental data [56]. Model adequacy and goodness-of-fit were further assessed using the CV, PRESS, and AP [57]. The obtained values demonstrate a strong agreement between the experimental and predicted responses, confirming the reliability and predictive capability of the developed models [58].

As presented in Table 4, the high coefficient of determination (R^2^ = 0.99), together with an adjusted R^2^ of 0.9772, signifies that 99% of the variability in Zn solubilization by *Serratia marcescens* AEYTU-11 is explained by the developed model, demonstrating its excellent predictive performance. The model’s adequacy and robustness are further confirmed by the tight agreement and near-unity values of the R2 statistics [59]. Low data variability and acceptable experimental precision are indicated by a coefficient of variation (C.V.) of 5.04%. Additionally, the corrected R^2^ and the projected R^2^ value of 0.9099 coincide rather well, suggesting high predictive power. The model’s capability for navigating the design space is confirmed by the adequate precision value of 28.48, which is significantly higher than the suggested minimum value of 4 and shows an adequate signal-to-noise ratio [60,61]. When taken as a whole, these statistical indicators confirm that the quadratic model accurately depicts the Zn solubilization by *Serratia marcescens* AEYTU-11 and may be consistently used for process prediction and optimization.

### 3.6. Interaction Between Influencing Factors

To analyze the response variability and determine the optimal settings for crucial process parameters to maximize Zn solubilization, 2D and 3D plots were developed. These graphs illustrate how the dependent variable (Zn solubilization) is influenced by the independent variables of initial insoluble Zn concentration, pH, and incubation duration. Two chosen independent variables are displayed on the X- and Y-axes of each 3D and 2D plot, while the Zn solubilization mg L^−1^ is displayed on the Z-axis. Throughout the plotting process, all other variables were maintained constantly at their central (coded) values.

#### 3.6.1. Effect of Initial Insoluble Zn Concentration and pH

**Figure 2:**
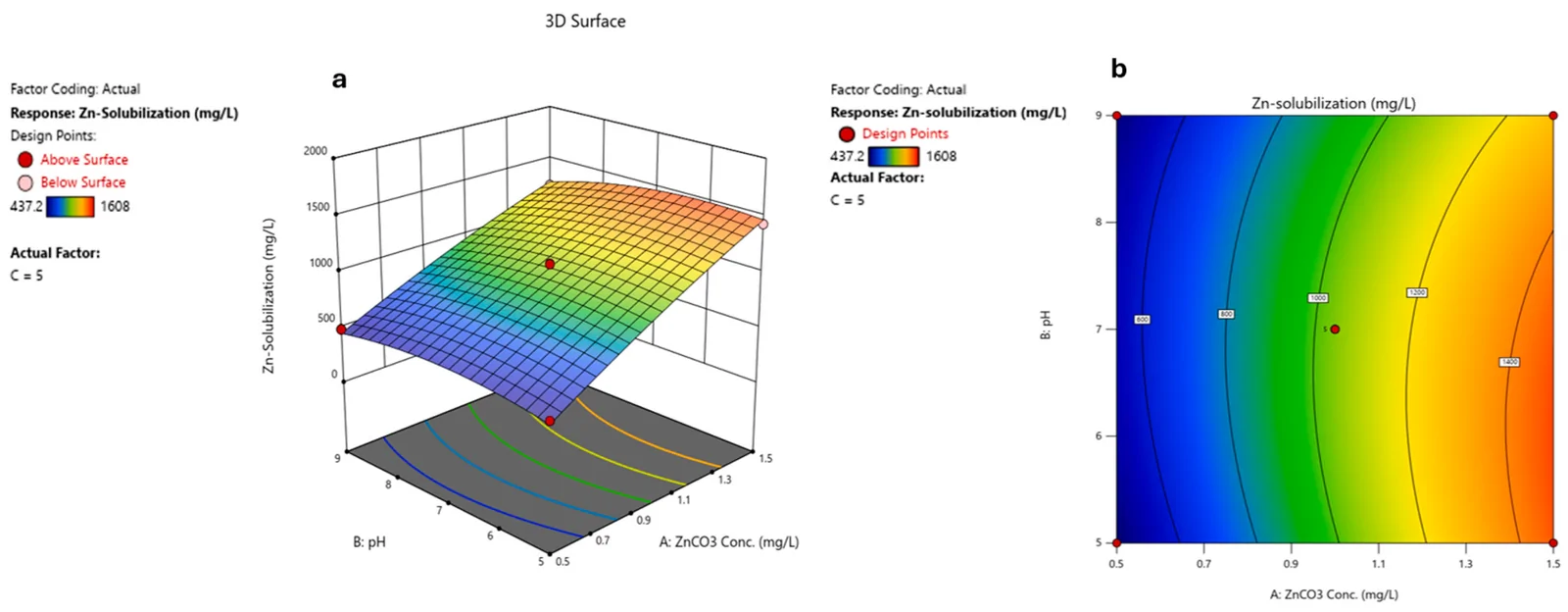
Three-dimensional (**a**) and contour (**b**) response surface plots showing the interactive effects of ZnCO_3_ concentration (A) and pH (B) on Zn-solubilization by *Serratia marcescens*.

Figure 2 illustrates the 3D and 2D plots, showing the influence of initial insoluble Zn (ZnCO_3_) content and pH on AEYTU-11’s ability to solubilize Zn. 3D plot revealed that ZnCO_3_ concentration exerted a positive effect on Zn solubilization, whereas pH had a negative influence. Zn solubilization was shown to increase with increasing ZnCO_3_ concentration, achieving maximum solubilization of 1431 ± 45 mg L^−1^, as the concentration increased from 0.5 to 1.5%. This indicates that substrate availability enhanced the solubilization process, and the *Serratia marcescens* AEYTU-11 strain can tolerate higher levels of ZnCO_3_ and continue its solubilization activity. However, the release of Zn^2+^ ions into the medium might become toxic to bacteria, affecting their development and decreasing the production of organic acids required for Zn solubilization, as the concentration of insoluble Zn compounds increases [25]. Therefore, the ability of bacterial isolates to withstand higher quantities of soluble Zn is necessary for persistent Zn solubilization.

pH has a crucial role in deciding how well solubilization works since it affects the ionic species in the solution. Throughout the investigation, we observed a significant decrease in the medium’s pH level (Table 3), which may have been brought on by the production of organic acids by AEYTU-11. However, raising the pH from 5 to 9 decreased Zn solubilization, most likely because of lower H^+^ availability and less ZnCO_3_ dissolution in alkaline environments. An interaction between ZnCO_3_ concentration and pH is indicated by the curved response surface and non-parallel contour lines (Figure 2b), with the influence of ZnCO_3_ concentration more pronounced at acidic pH values. Maximum Zn solubilization was attained at low pH and high ZnCO_3_ concentration, indicating that acidic environments promote ZnCO_3_ dissolution and the consequent release of soluble Zn ions. The bacteria’s synthesis and secretion of acids are responsible for the solubilization of insoluble Zn. By reducing the pH of the surrounding medium and promoting metal solubilization, these organic acids are essential for improving the solubilization of insoluble Zn complexes. The ensuing acidification increases the availability of Zn in the environment by encouraging the release of Zn ions from insoluble mineral complexes [3,62]. These results suggest that optimizing pH and ZnCO_3_ concentration is essential for optimizing Zn bioavailability and solubilization efficiency.

#### 3.6.2. Effect of Initial Insoluble Zn Concentration and Incubation Time

**Figure 3:**
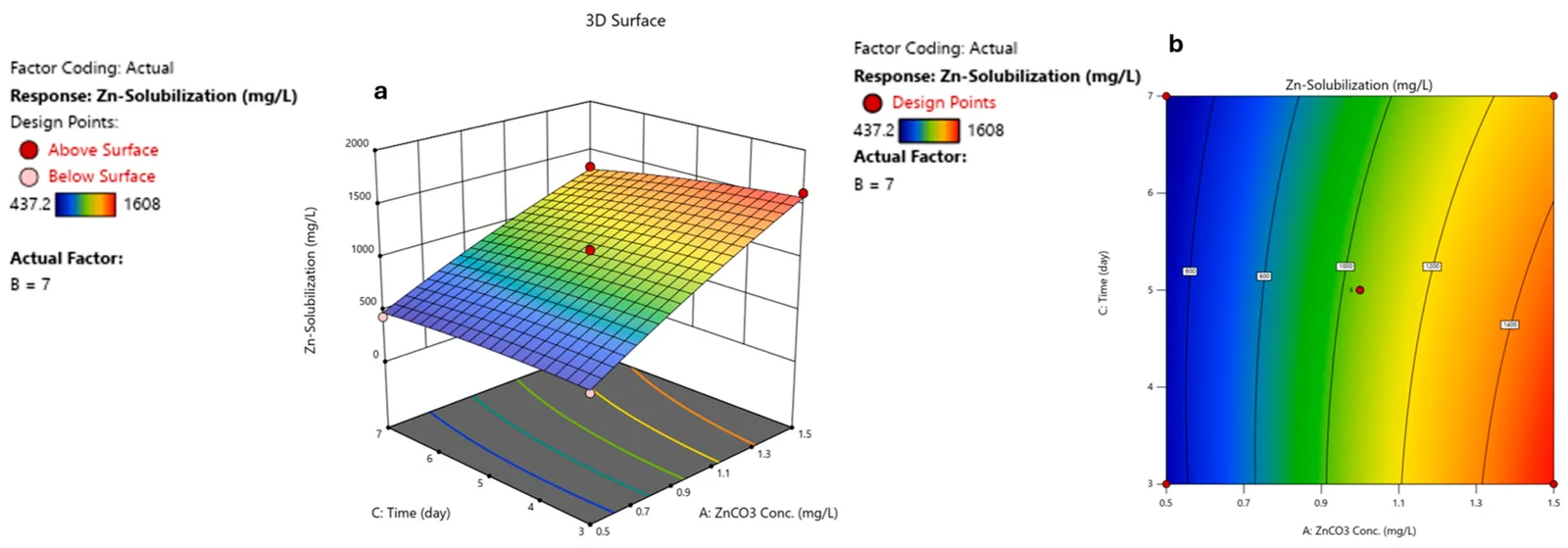
Three-dimensional (**a**) and contour (**b**) response surface plots showing the interactive effects of ZnCO_3_ concentration (A) and time (C) on Zn-solubilization by *Serratia marcescens*.

The combined effect of ZnCO_3_ content and incubation time on the AEYTU-11 strain’s Zn solubilization efficiency is depicted in Figure 3. The response surface analysis showed that whereas incubation time had a somewhat negative impact on Zn solubilization, ZnCO_3_ concentration had a substantial favorable effect. Zn solubilization increased progressively with increasing ZnCO_3_ concentration, from 0.5 to 1.5, and achieved a maximum solubilization of 1608 ± 51 mg L^−1^, indicating that substrate availability was a major determinant of Zn release. Conversely, prolonged incubation resulted in a slight reduction in soluble Zn concentration, possibly due to Zn re-precipitation, depletion of organic acids, or changes in microbial activity. However, Choudhary et al. [15] have observed that Zn solubilization increases with prolonged incubation time. The curvature of the response surface (Figure 3) suggests an interaction between ZnCO_3_ concentration and incubation time, whereby the positive effect of ZnCO_3_ concentration was more pronounced at shorter incubation periods. The highest Zn solubilization was predicted at elevated ZnCO_3_ concentration and lower incubation time, indicating that rapid dissolution of Zn carbonate occurred during the early stages of incubation before secondary immobilization processes became significant.

#### 3.6.3. Effect of pH and Incubation Time

**Figure 4:**
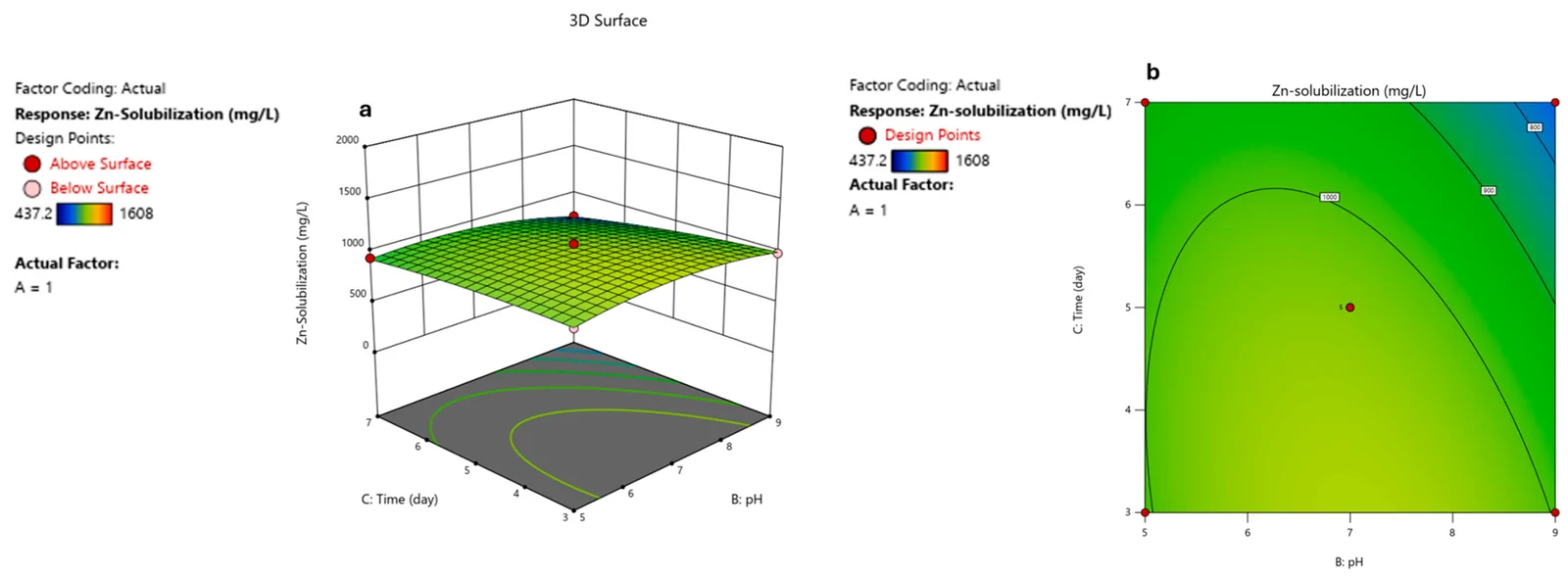
Three-dimensional (**a**) and contour (**b**) response surface plots showing the interactive effects of pH (B) and time (C) on Zn-solubilization by *Serratia marcescens*.

The response surface analysis (Figure 4a) revealed that both pH and incubation time significantly influenced Zn solubilization. Zn release increased with increasing incubation time and pH up to an optimum region, beyond which the response gradually declined. There are quadratic effects and a substantial interaction between pH and incubation duration, as seen by the dome-shaped response surface and elliptical contour lines (4b). Neutral pH and an intermediate incubation duration (5 days) produced the most Zn solubilization (1075 + 34 mg L^−1^), indicating that these circumstances promoted microbial growth and the generation of organic acids, which are responsible for the dissolving of ZnCO_3_. Reduced microbial activity, nutrient depletion, and re-precipitation of soluble Zn species may be the causes of the decrease in Zn solubilization seen during longer incubation times and higher pH levels. *Bacillus megaterium* ZnSB2’s Zn availability was shown to rise from 65.11 mg L^−1^ on day 3 to a peak of 126.80 mg L^−1^ on day 5 before falling to 54.57 mg L^−1^ by day 10 [3]. These results show that to enhance Zn bioavailability, pH and incubation duration must be tuned together.

## 4. Conclusions

The ability of the *Serratia marcescens* AEYTU-11 strain to solubilize the vital micronutrient Zn through an organic acid-mediated mechanism was demonstrated in this study. To predict the effects of different doses of an insoluble Zn compound (ZnCO_3_), pH, and incubation time on Zn solubilization by *Serratia marcescens*, experimental data were generated using the Box–Behnken Design (BBD) based Response Surface Methodology (RSM) approach. To maximize the Zn-solubilization capability of the *Serratia marcescens* AEYTU-11 strain in a laboratory setting, the constructed model enabled estimation of the optimal levels of the selected process parameters. ZnCO_3_ concentration was found to be the most significant factor, followed by pH and incubation duration, according to the analysis. Zn solubilization peaked at near-neutral pH and moderate incubation times, and it increased with increasing ZnCO_3_ concentration. Significant interactions between the variables were shown by response surface plots, underscoring the significance of concurrent process condition optimization. A substantial model F-value (77.21), a *p*-value of less than 0.0001, and a non-significant lack-of-fit test all supported the model’s adequacy and validity, according to ANOVA. Zn solubilization increased from 954 mg L^−1^ to 1608 mg L^−1^ after process parameters were optimized, a 1.69-fold improvement (68.6% increase) over the pre-optimization settings. Overall, improved Zn solubilization was the outcome of the adjusted circumstances, indicating the system’s potential to increase Zn bioavailability and aid in the creation of effective biofertilizer formulations for sustainable agriculture. Even though the AEYTU-11 strain showed promising results, more investigation is required to determine its efficacy in an agroecological setting.

## Figures and Tables

**Table 1:** Full factorial experimental design with factors and levels.

Variables	Symbol	Coded Levels		
		Low (−)	Middle (0)	High (+1)
ZnCO_3_ concentration (g/L)	A	0.5	1	1.5
pH	B	5	7	9
Time (day)	C	3	5	7
Response (objective)	Y	Maximize Zn solubilization (mg/L)		

**Table 2:** Zinc solubilization index (*ZnSI*) and Zinc solubilization efficiency (*ZnSE*) of bacterial strains under in vitro conditions.

Strain	Zn Source	Colony Diameter (HZ) (mm)	Halazone Diameter (HzD) (mm)	ZnSI	ZnSE (%)
AEYTU-11	ZnO	17	29	2.71	271
	ZnCO_3_	11	27	3.45	345
AEYTU-13	ZnO	10	16	2.60	260
	ZnCO_3_	9	12	2.33	233
AEYTU-15	ZnO	13	18	2.38	238
	ZnCO_3_	12	15	2.25	225

**Table 3:** BBD-based Zn-solubilization performance of AEYTU-11 with predicted and actual variables.

Run	Experimental Conditions			Response/Solubilization (mg L^−1^)		
	ZnCO_3_ Conc. (g^−1^) (A)	pH (B)	Time (Days) (C)	Predicted	Actual	pH Variation
1	1.5	7	3	1565.79	1608.00	3.49
2	0.5	9	5	449.52	485.70	5.69
3	1	7	5	1043.00	1082.00	2.93
4	1	7	5	1043.00	1076.00	2.83
5	1	7	5	1043.00	1075.00	2.85
6	1.5	9	5	1271.60	1240.00	5.62
7	1.5	7	7	1305.44	1331.00	3.59
8	1	5	7	926.99	937.60	2.91
9	1	7	5	1043.00	984.20	2.96
10	0.5	5	5	430.40	462.00	2.87
11	1	7	5	1043.00	997.80	2.84
12	1	9	7	747.46	753.50	5.56
13	0.5	7	3	532.96	507.40	2.96
14	1	9	3	995.71	985.10	5.56
15	0.5	7	7	479.41	437.20	2.89
16	1.5	5	5	1467.17	1431.00	3.15
17	1	5	3	992.64	986.60	3.30

**Table 4:** ANOVA and fit statistics for the response surface quadratic model for Zn-solubilization by AEYTU-11.

Source	Sum of Squares	df	Mean Square	F-Value	*p*-Value	
Model	1.878 × 10^6^	9	2.086 × 10^5^	77.21	<0.0001	significant
A-ZnCO_3_ Conc.	1.728 × 10^6^	1	1.728 × 10^6^	639.28	<0.0001	
B-pH	15,567.30	1	15,567.30	5.76	0.0475	
C-Time	49,266.60	1	49,266.60	18.23	0.0037	
AB	11,524.02	1	11,524.02	4.26	0.0778	
AC	10,691.56	1	10,691.56	3.96	0.0870	
BC	8335.69	1	8335.69	3.08	0.1225	
A^2^	7273.44	1	7273.44	2.69	0.1449	
B^2^	39,423.08	1	39,423.08	14.59	0.0065	
C^2^	3926.48	1	3926.48	1.45	0.2672	
Residual	18,917.69	7	2702.53			
Lack of Fit	9783.21	3	3261.07	1.43	0.3587	
Pure Error	9134.48	4	2283.62			
Cor Total	1.897 × 10^6^	16				
Std. Dev.	51.99		R^2^		0.9900	
Mean	963.54		Adjusted R^2^		0.9772	
C.V. %	5.40		Predicted R^2^		0.9099	
			Adeq Precision		28.4763	

## Data Availability

The original contributions presented in this study are included in the article. Further inquiries can be directed to the corresponding author.

## Supplementary Materials

The following supporting information can be downloaded at: https://www.mdpi.com/article/10.3390/microorganisms14102286/s1, Table S1: BLAST sequence alignment results; Table S2: Phenotypic, enzymatic and other functional traits of the AEYTU-11 strain; Figure S1: Zinc solubilization by AEYTU-11 on an agar plate supplemented with two different insoluble Zn sources (ZnO, ZnCO_3_); Figure S2. A neighbor-joining phylogenetic tree of the Zn-solubilizing AEYTU-11 strain and additional reference strains based on 16S rDNA gene sequence. MEGA 12 software was used to construct a bootstrap consensus tree by multiple sequence alignment using neighbor-joining; Figure S3. AEYTU-11 on nutrient agar.
