# Supplementary material for: Bioprocess Engineering for Enhanced Zinc Solubilization Through Response Surface Methodology (RSM) by Newly Isolated Rhizobacteria
Source: Microorganisms. 2026 Oct 8;14(10):2286. doi: 10.3390/microorganisms14102286 (PMC13707974; doi:10.3390/microorganisms14102286)

Bioprocess Engineering for Enhanced Zinc Solubilization Through RSM by Newly Isolated Rhizobacteria

Mohd Ashraf Dar [1,*], Ebru Koca Akkaya [1], Ommer Bhat [2], Mahdi Hassan [1] and Garima Kaushik [2]

[1] Department of Environmental Engineering, Yildiz Technical University, Davutpasa, Esenler, 34220, Istanbul, Türkiye

[2] Department of Environmental Science, School of Earth Sciences, Central University of Rajasthan, BandarSindri, Ajmer, Rajasthan 305817, India

Table S1. BLAST sequence alignment results

Description	Max Score	Query Cover	E-Value	Per. Identification	Accession No.
Serratia marcescens strain MH6	2760	100%	0	99.73%	FJ853424.1
Serratia marcescens strain HBQA7	2756	100%	0	99.73%	PQ327582.1
Serratia marcescens strain TP1	2756	100%	0	99.73%	MW879529.1
Serratia marcescens strain P3	2756	100%	0	99.73%	MZ477005.1
Serratia marcescens strain WES1	2756	100%	0	99.73%	MN960115.1
Serratia marcescens strain CS2101	2756	100%	0	99.73%	KY379045.1
Serratia marcescens strain NRC408	2756	100%	0	99.73%	OR793165.1
Serratia marcescens strain KMJ-03	2756	100%	0	99.67%	PX482431.1
Serratia marcescens strain KRED	2756	100%	0	99.73%	NR_036886.1
Serratia marcescens strain Gol3	2756	100%	0	99.73%	MT263018.1
Serratia marcescens strain VMG14	2756	100%	0	99.73%	MN636438.1
Serratia marcescens strain GMB S5	2756	100%	0	99.73%	OR974900.1
Serratia marcescens strain DSIP-2	2754	100%	0	99.73%	KJ631129.1

Table S2. Phenotypic, enzymatic and other functional traits of the AEYTU-11 strain.

Test	Result
Gram reaction	Gram negative
Shape	Rod
KOH stirring	+
motility	+
Spore formation	-
Hemolysis	Gamma
H_2S production	-
Indole	-
Urease	-
Methyl red	-
Lactose	+
Citrate	+
Catalase	+
IAA	-
HCN	+
α-amylase,	-
Cellulase	+
Protease	+
P solubilization	-
K solubilization	+

Figure S1. Zinc solubilization by AEYTU-11 on an agar plate supplemented with two different insoluble Zn sources (ZnO, $ZnCO_3$)

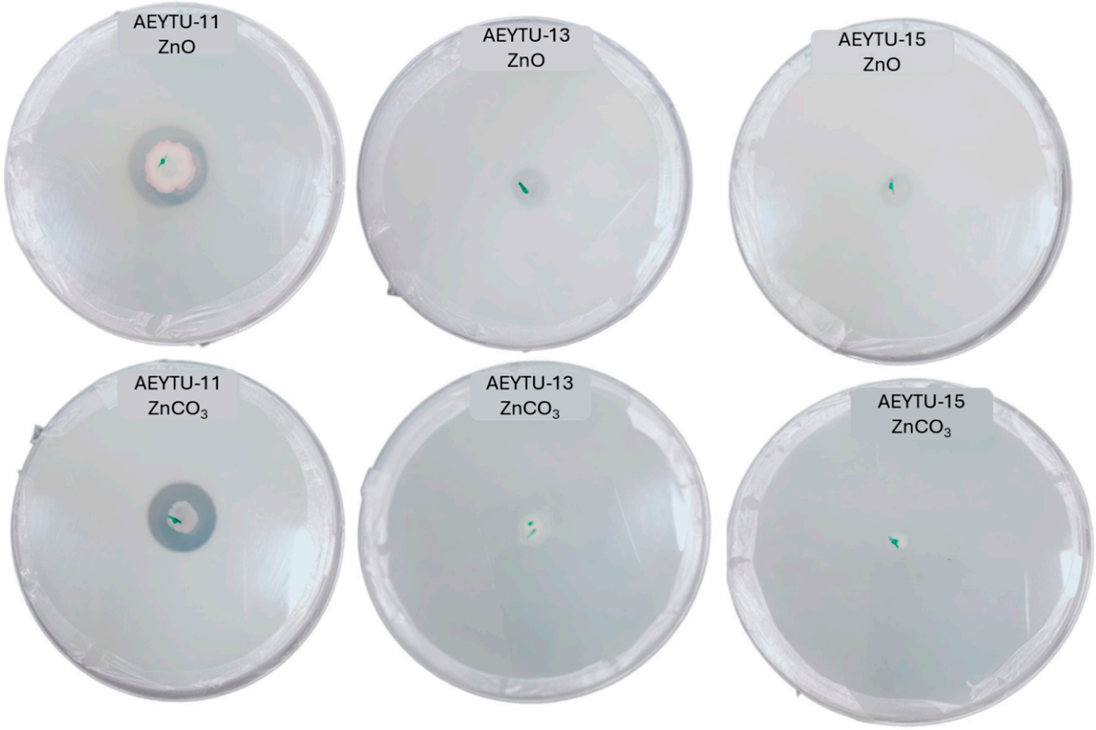

Figure S2. A neighbor-joining phylogenetic tree of the Zn-solubilizing AEYTU-11 strain and additional reference strains based on 16S rDNA gene sequence. MEGA 12 software was used to construct a bootstrap consensus tree by multiple sequence alignment using neighbor-joining.

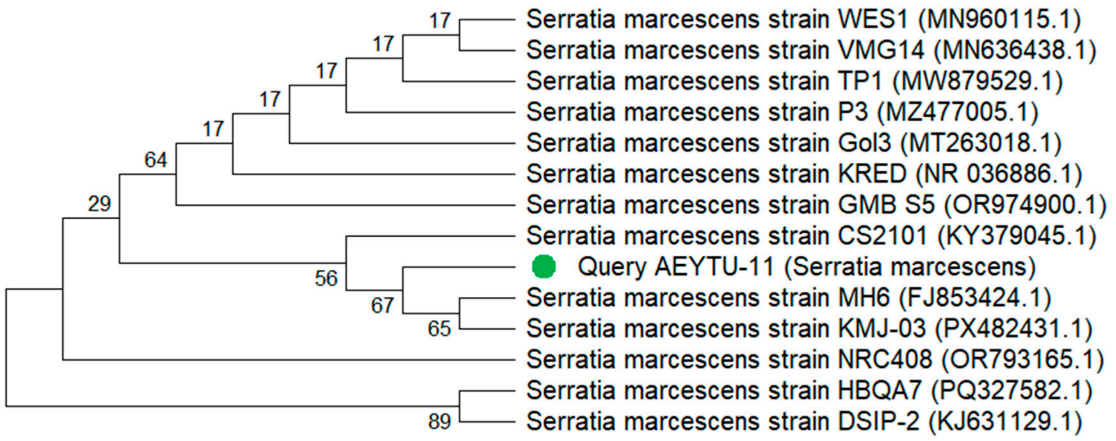

Figure S3. AEYTU-11 on nutrient agar.

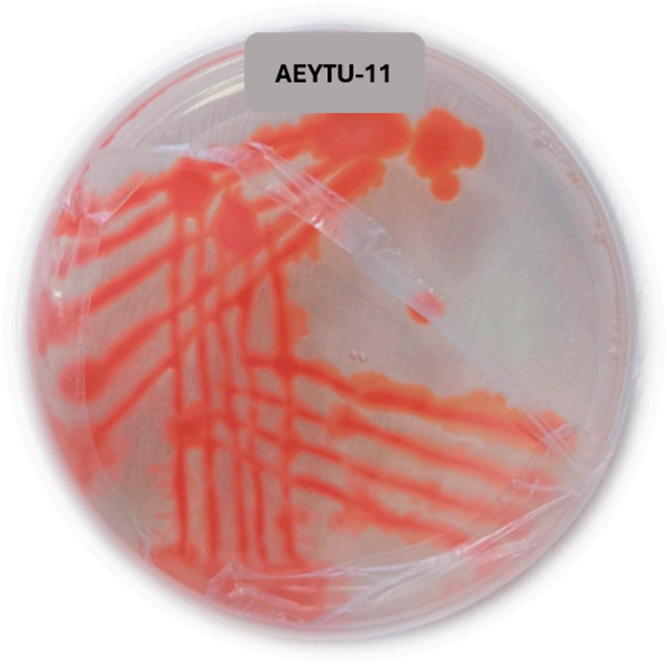

Supplement: Supplementary file 1 [file microorganisms-14-02286-s001.zip › microorganisms-4573478-supplementary.pdf]
